# Epidemic trend analysis of SARS‐CoV‐2 in South Asian Association for Regional Cooperation countries using modified susceptible‐infected‐recovered predictive model

**DOI:** 10.1002/eng2.12550

**Published:** 2022-07-10

**Authors:** Samrat Kumar Dey, Md. Mahbubur Rahman, Kabid Hassan Shibly, Umme Raihan Siddiqi, Arpita Howlader

**Affiliations:** ^1^ School of Science and Technology (SST) Bangladesh Open University (BOU) Gazipur Bangladesh; ^2^ Department of Computer Science and Engineering (CSE) Military Institute of Science and Technology (MIST) Dhaka Bangladesh; ^3^ Laboratory for Cyber Resilience Nara Institute of Science and Technology (NAIST) Nara Japan; ^4^ Department of Physiology Shaheed Suhrawardy Medical College (ShSMC) Dhaka Bangladesh; ^5^ Department of Computer and Communication Engineering (CCE) Patuakhali Science and Technology University (PSTU) Dumki Patuakhali Bangladesh

**Keywords:** COVID‐19, epidemics, modified susceptible‐infected‐recovered, prediction model, SAARC, SARS‐CoV‐2

## Abstract

A novel coronavirus causing the severe and fatal respiratory syndrome was identified in China, is now producing outbreaks in more than 200 countries around the world, and became pandemic by the time. In this article, a modified version of the well‐known mathematical epidemic model susceptible‐infected‐recovered (SIR) is used to analyze the epidemic's course of COVID‐19 in eight different countries of the South Asian Association for Regional Cooperation (SAARC). To achieve this goal, the parameters of the SIR model are identified by using publicly available data for the corresponding countries: Afghanistan, Bangladesh, Bhutan, India, the Maldives, Nepal, Pakistan, and Sri Lanka. Based on the prediction model, we estimated the epidemic trend of COVID‐19 outbreak in SAARC countries for 20, 90, and 180 days, respectively. A short‐mid‐long term prediction model has been designed to understand the early dynamics of the COVID‐19 epidemic in the southeast Asian region. The maximum and minimum basic reproduction numbers (*R*_0_ = 1.33 and 1.07) for SAARC countries are predicted to be in Pakistan and Bhutan. We equate simulation results with real data in the SAARC countries on the COVID‐19 outbreak, and predicted different scenarios using the modified SIR prediction model. Our results should provide policymakers with a method for evaluating the impacts of possible interventions, including lockdown and social distancing, as well as testing and contact tracking.

## Graphical abstract

1. An short‐mid‐long term prediction model has been designed to understand the early dynamics of the COVID‐19 epidemic in the southeast Asian region.
2. Based on the proposed prediction model we estimated the epidemic trend of COVID‐19 outbreak in SAARC countries for 20 days (short term), 90 days (mid‐term), and 180 days (long term), respectively.
3. Modified susceptible‐infected‐recovered model is used to calculate the transmission rate and recovery rate of each country in the SAARC regions.

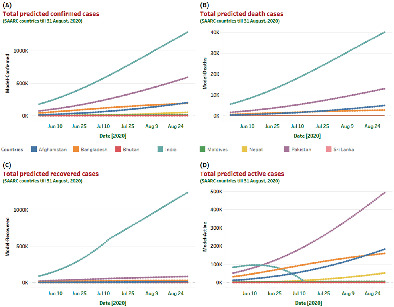

## 1 INTRODUCTION

A novel coronavirus (SARS‐CoV‐2), called COVID‐19, caused an outbreak in the city of Wuhan, Hubei Province, China that is linked to the Huanan Seafood Wholesale Market^1^, ^2^ in late December 2019. Till now, more than 200 countries around the world have been infected by a novel coronavirus. As of May 30, 2020, according to the World Health Organization (WHO), globally 5,817,385 confirmed cases have reported with a death count of 362,705.^3^ Among them, the south‐east Asia region has confirmed 4.2% of cases while the case fatality ratio is 2.86%. The very first COVID‐19 patient was treated for being coronavirus positive in Wuhan, China, at the beginning of December 2019. In the south‐east Asia region, the very first coronavirus infected patient was detected in Pakistan on February 26, 2020.^4^ On January 30, 2020, WHO officially declared this outbreak of COVID‐19 as global pandemic.^5^ To mitigate the spread of COVID‐19, the affected countries of the world have taken various measures, including citywide lockdown, social distancing, traffic halt, community management, and information on health education knowledge. More importantly, the outbreak of COVID‐19 possessed a massive threat to global health and economics all over the world. One of the significant feature of novel coronavirus unlike other infectious diseases like severe acute respiratory syndrome (SARS), and Middle East respiratory syndrome (MERS), it causes asymptomatic infections (symptoms are very mild).^6^, ^7^, ^8^ Responses to COVID‐19 in South Asian Association for Regional Cooperation (SAARC) nations in 2020 were reported and examined with available data in a recent study by Malik et al.^9^ For the months of April 2020 to December 2020, the authors utilized the exponential growth approach to calculate the reproductive number *R*_t_. Different epidemiological model parameters were also used to establish a correlation with the COVID‐19 transmission pattern. According to this study, immunization and accurate advice on COVID‐19 prevention measures may assist to reduce the risk of infection. Similar work has been proposed by Malek and Hoque^10^ in the domain of COVID‐19 epidemic trend analysis. The COVID‐19 trend in South Asian countries was explored by the authors using the SEIATR model, an upgraded version of the SEIR system. However, their recommended results only anticipated the COVID cases until June 2020 and only for four nations including India, Pakistan, Bangladesh, and Afghanistan. In another study, Tiwari et al.^11^ provided India‐centric mathematical modeling that focused on the repercussions of lockdown in another prediction of COVID‐19 disease spread. By studying nationwide lockdown in India, the authors used a SEIRD model with five compartments. Overall, the article argued that a global countrywide lockdown is an effective control policy for COVID‐19 spread. For analyzing the COVID‐19 spread across India, the authors in Reference 12 have presented a regression analysis model using the dataset available at Kaggle. However, the developed models is only capable of forecasting the next 6 days of COVID‐19 spread data and among the six utilized model, sixth‐degree polynomial regression outperformed others utilized models. In another study, the authors in Reference 13 used ML‐guided piecewise linear regression to predict COVID‐19 instances across India. The trend of COVID‐19 instances climbed linearly, then exponentially, according to the findings. However, only the six separate states of India were considered for the next 50 days in the projection of cases. In order to estimate the spread of COVID‐19 cases in India, the authors in Reference 14 suggested a deep LSTM architecture and attained an accuracy of 97.59% utilizing WHO data for India. Moreover, an extensive review of the available worldwide datasets for COVID‐19 has been reviewed thoroughly. The authors in Reference 15 provide a time‐dependent based susceptible‐infected‐recovered (SIR) model that is capable of detecting transmission and recovery rates using data provided by China. This research also proposed two distinct ways to social separation in order to minimize the effective reproduction number. This study only emphasized eight different world counters in five separate projection categories, including the United States, the United Kingdom, France, Iran, Spain, Italy, Germany, and the Republic of Korea. In these circumstances, the rate of transmission among a large number of people can increase within no time. According to the latest World Health Organization survey, only 87.9% of COVID‐19 patients have a fever, and 67.7% have dry cough.^16^ Therefore, this is highly crucial to estimate the intensity of the COVID‐19 epidemic and predict the time course, peak time, total duration, and so on. In recent times, the authors in Reference 17 have investigated and projected COVID‐19 instances using three distinct models (SIR, SEIQR, and ML), concentrating on three different nations (Australia, the United Kingdom, and the United States). The research found that the Prophet Algorithm fared better in the UK and the United States than in Australia among the ML models. However, to the best of our knowledge, no previous research has been conducted to predict COVID‐19 instances focused on countries in the South Asian region. Therefore, our study focuses on the COVID‐19 case prediction using a modified SIR (M‐SIR) model in the countries of the SAARC. SAARC is considered as an intergovernmental organization and geopolitical union of states in South Asia. Its member states are Afghanistan, Bangladesh, Bhutan, India, the Maldives, Nepal, Pakistan, and Sri Lanka. Our aim is to develop a prediction model for the SAARC countries to understand the epidemiological trend of novel coronavirus outbreak in these countries. Here we explore a modified version based on the SIR epidemic model to predict the short term (20 days), mid‐term (90 days), and long term (180 days) evaluation of COVID‐19 situation in these countries of SAARC regions.

## 2 METHODOLOGY

The research methodology is divided into several phases. First, appropriate data were collected from four different data sources including Johns Hopkins University (JHU), COVID‐19 Dataset available at Kaggle, COVID‐19 Open Research Dataset, and population by country 2020 dataset. The data were then evaluated and preprocessed to eliminate any duplicate or missing values. This section also includes the working principle of M‐SIR model, and then discussed the proposed algorithm of this research.

### 2.1 Dataset analysis

For this exploration, we have used datasets from various sources for our analysis and building the model. We have used four different sources of the dataset including, COVID‐19 Data Repository by the Center for Systems Science and Engineering (CSSE) at Johns Hopkins University (January–May 2020),^18^ COVID‐19 Dataset (January–May 2020),^19^ COVID‐19 Open Research Dataset (CORD‐19),^20^ and population by country 2020 dataset.^21^ Table 1 provides insight on each dataset and their respective data files with their column description.

**TABLE 1:** Tabular representation of different data sources of COVID‐19

Dataset	Description	Columns
*Novel Coronavirus 2019 Dataset*		
COVID19_line_list_data.csv	This file is an aggregated version of the Novel Coronavirus dataset collected by Johns Hopkins University	Id, case_in_country, reporting date, summary, location, country, gender, age, symptom_onset, If_onset_approximate, hosp_visit_date, exposure_start, exposure_end, visiting Wuhan, from Wuhan, death, recovered, symptom, source
covid_19_data.csv	Daily level information on the number of COVID‐19 affected cases across the globe	Sno, observation date, province/state, country/region, last update, confirmed, deaths, recovered
time_series_covid_19_confirmed.csv	Time series data on the number of confirmed cases	Province/state, country/region, lat, long, 1/22/20…0.5/6/20
time_series_covid_19_deaths.csv	Time series data on the number of death cases	Province/state, country/region, lat, long, 1/22/20…0.5/6/20
time_series_covid_19_recovered.csv	Time series data on the number of recovered cases	Province/state, country/region, lat, long, 1/22/20…0.5/6/20
*COVID‐19 (nCoV‐19) Coronavirus Spread Dataset*		
covid_19_clean_complete.csv	The file contains the cumulative count of confirmed, death and recovered cases of COVID‐19 from different countries from January 2020	Province/state, country/region, lat, long, date, confirmed, deaths, recovered
*COVID‐19 Open Research Dataset*		
metadata.csv	This dataset contains metadata for 59k articles on COVID‐19	Cord_uid, sha, source_x, title, doi, pmcid, license, abstract, publish_time, authors, journal
*Population by country 2020 dataset*		
population_by_country_2020.csv	This dataset contains the information from 235 countries along with their population till 2020	Country (or dependency), population (2020), yearly change, net change, density (P/km^2^), land area (km^2^), migrants (net), fert. rate, med. age, urban pop %, world share

We also developed a custom dataset to develop and evaluate our model. Table 2 is developed based on collected data from different sources.

**TABLE 2:** Columns description of custom build COVID‐19 dataset

Field name	Field data description
Sno.	Serial number
Province/state	Province or state of the observation
Country/region	Country of observation
Date	Date and time of the observation in MM/DD/YYYY HH:MM: SS
Confirmed	Number of confirmed cases
Deaths	Number of deaths
Recovered	Number of recovered cases
Active	Number of active cases
1/22/20	First reporting date
5/18/2020	Latest reporting date

### 2.2 M‐SIR model

Predictive mathematical disease models are important for understanding the trajectory of the outbreak and for preparing successful response strategies. One commonly used model is the human‐to‐human transmission SIR model, which defines people's flow through three mutually exclusive stages of infection: susceptible (S), infected (I), and recovered (R). Most epidemic models are based on dividing the population into a small number of sections. Each person is identical in terms of their status with the considered disease. The SIR model based on three sections: susceptible (*S*) is the class for those who are susceptible to infection. This can include passive immune systems as soon as they lose their immunity. In the infected (*I*) class, the parasite level within the host is large enough, and there is a possibility of spreading the infection to other susceptible people. The recovered (*R*) class includes all infected and recovered individuals. This epidemiological model captures the dynamics of acute infections that, after recovery, confer lifelong immunity. In general, the overall size of the population is considered constant *N* = *S* + *I* + *R*. The two cases should be examined and characterized by the inclusion or exclusion of demographic factors. Let us assume in the SIR model; there is a natural host lifespan of 1/*μ* years. Then the rate at which individuals in an epidemiological compartment suffer from natural mortality is given by *μ*. It is important to emphasize that this factor is independent of the disease and should not reflect the pathogenicity of the infectious agent. Diachronically, it can be expressed in Equation (1).

(1) $$\frac{\mathrm{dS}}{\mathrm{dt}}+\frac{\mathrm{dI}}{\mathrm{dt}}+\frac{\mathrm{dR}}{\mathrm{dt}}=0.$$

So, the SIR model can be defined using Equation (2),

(2) $$\left\{\begin{array}{c} \frac{\mathrm{dS}}{\mathrm{dt}}=\mu -\beta \mathrm{SI}-\mu S,\\ \frac{\mathrm{dI}}{\mathrm{dt}}=\beta \mathrm{SI}-\gamma I-\mu I,\\ \frac{\mathrm{dR}}{\mathrm{dt}}=\gamma I-\mu R.\; \end{array}\right.$$

Here, the initial conditions *S*(0) > 0, *I*(0) ≥ 0, and *R*(0) ≥ 0. It is important to enter the expression of basic reproduction number *R*_0_ for this model. The basic reproduction number *R*_0_ is the parameter that estimates whether a disease has spread to the population or not. If the estimated *R*_0_ < 1, we can assume that the disease will die out, and if *R*_0_ = 1, the disease remains in the system and is stable. But if *R*_0_ > 1, the disease will spread and cause an outbreak. The higher the value of *R*_0_, the more difficult it is to control.

### 2.3 Proposed model and algorithm

**FIGURE 1:**
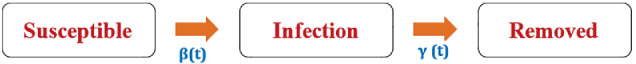
Modified SIR (M‐SIR) model with the time‐varying transmission. It can dynamically adjust the crucial parameters while working on time‐varying data, which is also treated as an M‐SIR model.

In this exploration, we have used an SIR epidemic model. In a general SIR model, transmission rate (*β*) and recovery rate (*γ*) are considered as two time‐invariant variables. Moreover, several research studies have shown that a SIR model works much better in presenting the information contained in the confirmed case data than an SEIR model.^22^ Therefore we have developed a model presented in Figure 1 that can dynamically adjust the crucial parameters while working on time‐varying data, which is also treated as an M‐SIR model.^23^ However, in a basic SIR model, the reproduction number ($R_{0}$) is a simple division of transmission and recovery rates, as shown in Equation (3).

(3) $$R_{0}=\frac{\beta }{\gamma +\mu }.$$

For building the model, we modified the primary reproduction number, $R_{0}$ with respect to time (*t*). Equation (4) represents the changes that happened depending on time (*t*).

(4) $$R_{0}\left(t\right)=\frac{\beta \left(t\right)}{\gamma \left(t\right)+\mu }.$$

We also considered detectable ($\rho_{1}$) and nondetectable ($\rho_{2}$) infected persons for building our model effectively, as shown in Equation (5).

(5) $$R_{0}\left(t\right)=\rho_{1}\frac{\beta {\left(t\right)}_{1}}{\gamma {\left(t\right)}_{1}}+\rho_{2}\frac{\beta {\left(t\right)}_{2}}{\gamma {\left(t\right)}_{2}}.$$

In general, detectable persons contain a lower transmission rate than nondetectable persons. Therefore we calculated the transmission rate (*β*) and recovery rate (*γ*) for each country of the SAARC region. For instance, Bangladesh has 165 million people,^24^ and the first confirmed case reported in the country was on March 7, 2020. As of May 30, 2020, a total of 42,844 COVID‐19 infected people were detected in the country.^25^ Depending on the M‐SIR model, Bangladesh contains a transmission rate of 0.63 with a recovery rate of 0.49. The initial reproduction number for the country is 1.27. Based on available data sources of every SAARC country, we have calculated these parameters for the prediction model, which is shown in Table 3. Algorithm 1 represents the working procedure of our proposed (M‐SIR) prediction model.

**TABLE 3:** Transmission rate, recovery rate, and reproduction number of SAARC countries

Countries	Transmission rate (*β*)	Recovery rate (*γ*)	Reproduction number (*R*_t_)
Afghanistan	0.5997691	0.4889147	1.22673566575
Bangladesh	0.6286615	0.4932285	1.2745847006
Bhutan	0.5334682	0.4982385	1.07070850607
India	0.5060977	0.3955793	1.27938367857
Maldives	0.5774223	0.4920300	1.17355100299
Nepal	0.5406592	0.4954823	1.09117762632
Pakistan	0.5254487	0.3947732	1.33101411139
Sri Lanka	0.5595021	0.4933888	1.13399838018

Algorithm 1 **M‐SIR prediction model**

1. Evaluate the transmission rate β(t), recovery rate γ(t), within the dataset time period, *T*
2. Train multiple regression or ridge regression
3. Estimate number of an infected person after the dataset time period, *T*
4. Estimate number of a recovered person after the dataset time period, *T*
5. **while** dataset time period, *T* ≤ respective time, *t* ≤ estimated time for prediction **do**
6. Estimate predicted transmission rate and recovery rate
7. Predict the number of affected persons, the number of recovered persons, the number of active cases
8. **end while**

The transmission rate *β* and the recovery rate *γ* are two time‐invariant variables in the conventional SIR model. *β* denotes that each person has on average *β* contacts with randomly selected others per unit of time. The recovery rate *γ*, on the other hand, suggests that diseased individuals recover or die at a constant average rate *γ*. As the time‐varying feature of *β* and *γ* is ignored in the traditional SIR model, therefore, this assumption is too simplistic to accurately and effectively anticipate the disease's progression.^15^ For this reason, to make the model robust, time‐dependent SIR (M‐SIR) model is proposed, in which both the transmission rate *β* and the recovery rate *γ* are functions of time *t*. Therefore, this type of M‐SIR model is far more effective at predicting disease spread, control, and future trends.

## 3 RESULTS

**FIGURE 2:**
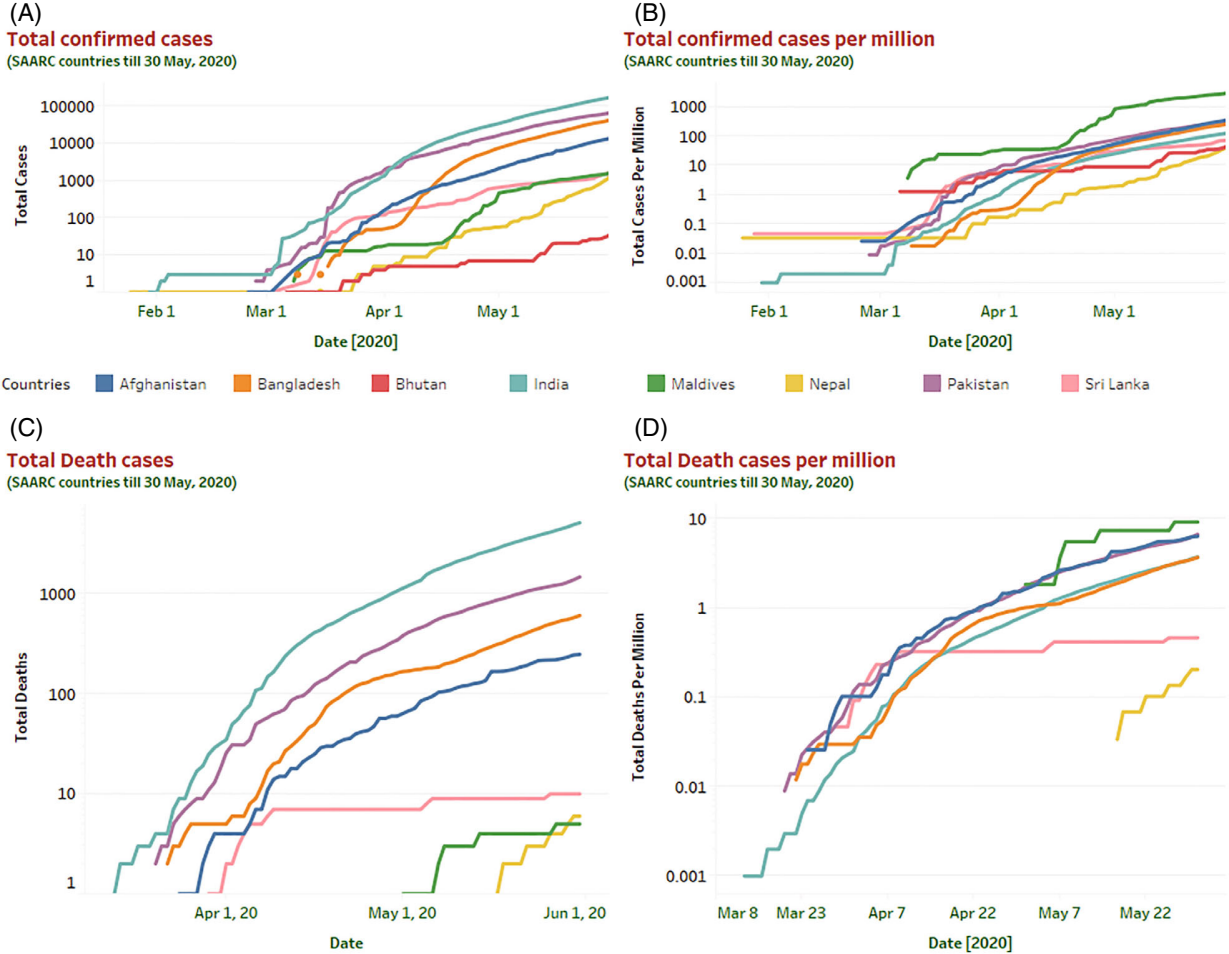
Graphical representation of countrywide COVID‐19 total confirmed and death cases along with the total number of confirmed and death cases per million till May 30, 2020. This graphical representation provides a comprehensive overview of the total number of confirmed and death cases in SAARC regions. Countrywise COVID‐19 confirmed and death cases along with the number of cases per million illustrated in (A–D) till May 30, 2020. Based on the illustration in (A) and (C), India has confirmed the highest number of infected cases (173,763) along with the most number of death cases (5164) in the region.

We performed our simulations and tabulated the predicted results (short term, midterm, and long term) with all countries from the SAARC region. We also explored 3D parameter space using gradient descent to minimize the error. However, a lag parameter is also used in this experiment to reduce the gap of first confirmed cases of SAARC countries' pandemic situation. The M‐SIR model was calculated using MATLAB R2020a software, and Tableau data analysis visualization tools were utilized to present the estimated data in a more dynamic manner. Table 3 represents the transmission rate (*β*), recovery rate (*γ*), and the reproduction numbers (*R*_t_) of all countries in the SAARC regions. Bangladesh contains the highest transmission rate of (*β* = ∼0.63) in the SAARC region, whereas India shows a much lower transmission rate of only ∼0.51 compared with other countries. Also, data analysis visualization tools have been employed (Figure 2) to understand the current (as of May 30, 2020) scenario of the countries (Afghanistan, Bangladesh, Bhutan, India, Maldives, Nepal, Pakistan, and Sri Lanka) in terms of their confirmed and death cases ratio.

**FIGURE 3:**
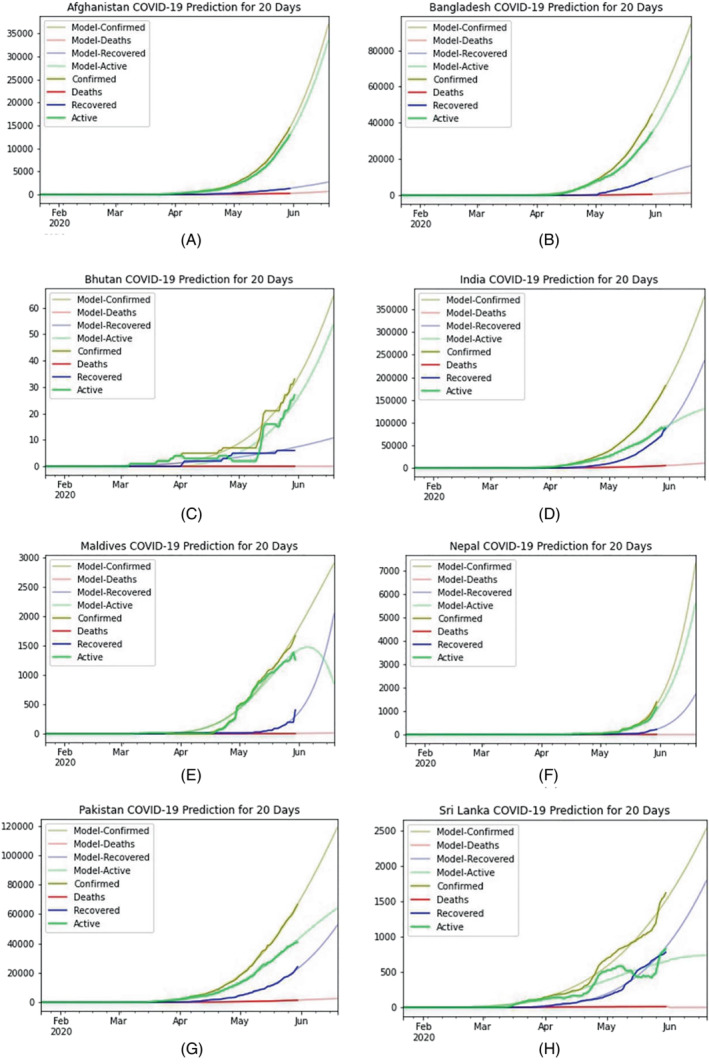
Graphical representation of a short‐term prediction curve till June 19, 2020, for all the countries of SAARC regions. This short‐term prediction model for the next 20 days provides a depiction of all SAARC countries' evaluation of COVID‐19 till mid‐June. All the countries prediction curves are denoted from (A–H), respectively, where (A) Afghanistan, (B) Bangladesh, (C) Bhutan, (D) India, (E) Maldives, (F) Nepal, (G) Pakistan, (H) Sri Lanka predicted the epidemiological curve for next 20 days successfully.

Surprisingly the slow growth of infections in the South‐Asian region could be a result of a lower number of testing and testing strategy.^26^ Initially, the testing was limited to specific individuals who have come from high‐risk countries. Even their immediate contacts were also ignored primarily. The initial growth rate of COVID‐19 infection in the SAARC region is comparatively lower than countries like the US, France, Germany, Spain, China, Italy, and so on. However, based on the current scenario in the SAARC region (as of May 30, 2020), India confirmed the highest number of COVID‐19 reported cases. A short term model prediction for the next 20 days (till June 19, 2020) is illustrated in Figure 3 for all the countries of SAARC regions. Similarly, we also predicted the epidemic curve of SAARC regions for the midterm (90 days) and long term (180 days) COVID‐19 cases. After predicting the 20 days of the epidemic, we noticed that India, Bangladesh, Sri Lanka, and Pakistan are increasing gradually by minimizing the active cases, and their recovery rate is also rising over time. However, in this short period, the confirmed cases of these countries are also increasing till June 19, 2020. A comprehensive statistical analysis for the long‐term prediction model has been developed for each country of the SAARC region. To highlight the results, this research considered the different descriptive statistical measures including mean, median, standard error, standard deviation, range, min value, max value, and different quartile values. Based on the prediction, Afghanistan, Bangladesh, India, and Pakistan are being considered the most severely affected region due to the adverse effect of COVID‐19 (Table 4).

**TABLE 4:** Statistical summary of 180 days prediction model for the most affected countries in the SAARC region

	Countries	Model‐confirmed	Model‐deaths	Model‐recovered	Model‐active
Mean	AFG	190,412.40	4643.50	11,126.18	174,642.719
	BAN	155,231.18	2218.13	23,731.48	129,281.56
	IND	211,200.72	3017.89	27,867.53	180,315.3
	PAK	528,468.79	11,653.97	68,238.28	448,576.53
Standard error	AFG	11,206.57	273.28	509.94	10,430.84
	BAN	4659.14	66.57	456.77	4168.88
	IND	47,359.55	1479.88	48,017.68	2700.96
	PAK	25,914.54	571.47	1998.50	23,473.099
Standard deviation	AFG	150,351.97	3666.56	6841.61	139,944.42
	BAN	62,509.03	893.20	6128.31	55,931.46
	IND	635,395.08	19,854.72	644,224.84	36,237.26
	PAK	347,680.15	7667.16	26,812.71	314,924.67
Range	AFG	485,970.31	11,851.13	21,504.85	452,614.31
	BAN	200,118.47	2859.53	22,442.97	174,815.96
	IND	2,004,354.33	62,631.73	2,004,242.36	94,846.56
	PAK	1,119,327.13	24,683.79	86,381.49	1,008,261.84
Minimum	AFG	7898.49	192.61	924.35	6781.52
	BAN	26,187.94	374.20	5558.10	20,255.63
	IND	110,541.12	3454.17	44,567.18	0
	PAK	46,959.35	1035.56	13,124.96	32,798.82
Maximum	AFG	493,868.81	12,043.75	22,429.20	459,395.84
	BAN	226,306.41	3233.74	28,001.08	195,071.59
	IND	2,114,895.46	66,085.91	2,048,809.55	94,846.56
	PAK	1,166,286.48	25,719.35	99,506.45	1,041,060.66
First quartile (25%)	AFG	51,363.72	1232.90	4631.15	44,692.82
	BAN	104,429.75	1492.22	21,777.67	81,159.87
	IND	494,619.83	15,455.8	427,771.65	0
	PAK	202,699.9	4470.01	46,338.15	151,891.8
Median (50%)	AFG	156,590.5	3818.70	10,947.42	141,824.4
	BAN	174,802.19	2497.78	26,986.22	145,318.2
	IND	1,095,738.02	34,239.44	1,061,498.57	0
	PAK	487,562.5	10,751.9	76,114.45	400,696.1
Third quartile (75%)	AFG	314,807	7724.63	17,425.18	291,608.2
	BAN	211,200.72	3017.89	27,867.53	180,315.3
	IND	1,679,552.22	52,482.38	1,621,471.80	59,168.73
	PAK	835,283.1	18,419.96	92,331.4	724,531.8

*Note*: Count: 180.

## 4 DISCUSSION

The US, Italy, Spain, Germany, China are the top most affected countries in the world due to the pandemic of COVID‐19. Though the novel coronavirus originated and started to spread from China but, countries from the South‐Asian region are also infected faster than other countries globally. The overall population density of the countries in the SAARC regions is also too high. According to Table 5, we can observe the various parameters (population, land area, density, and world share) and their impact on analyzing the spread of novel coronavirus in these regions.

**TABLE 5:** Population, total area, and density of SAARC countries with their individual world share^21^

Countries	Population	Land area (km^2^)	Density (P/km^2^)	World share %
Afghanistan	38,742,911	652,860	60	0.50
Bangladesh	164,354,176	130,170	1265	2.11
Bhutan	769,867	38,117	20	0.01
India	1,377,233,523	2,973,190	464	17.70
Maldives	538,558	300	1802	0.01
Nepal	29,027,347	143,350	203	0.37
Pakistan	219,992,900	770,880	287	2.83
Sri Lanka	21,395,196	62,710	341	0.27

We further extended our prediction model for the next 90 days and 180 days, respectively. Besides, the total number of the population of a country is also parameterized because the total population of a country cannot be infected. Therefore, if we consider the total population as infected, then the probable number of infected persons remains unknown. Based on the prediction model, we tabulated the model predicted results of all SAARC countries for short term, midterm, and long term prediction, respectively (Table 6).

**TABLE 6:** Predicted data based on M‐SIR model for the countries of SAARC region

Countries		Short term prediction (20 days)	Mid‐term prediction (90 days)	Long term prediction (180 days)	Remarks
Afghanistan	Confirmed	35,286	297,264	1,092,714	Confirmed, recovery and active cases will increase at each predictive term In the long‐term forecast, the number of death cases will decrease comparing with the mid‐term forecast
	Deaths	652	5498	3521	
	Recovered	2555	8521	13,416	
	Active	32,078	283,245	1,075,775	
Bangladesh	Confirmed	100,037	451,840	867,700	Significant rise will be observed in confirmed and active cases The number of recovered cases after mid‐term prediction will not increase much in the long‐term prediction
	Deaths	1346	6080	11,676	
	Recovered	17,724	29,418	30,476	
	Active	80,966	416,341	825,547	
Bhutan	Confirmed	61	372	1683	Large number of confirmed cases but not enough recovery The number of deaths will remain zero
	Deaths	0	0	0	
	Recovered	11	32	79	
	Active	50	340	1603	
India	Confirmed	378,657	1,717,831	3,709,965	The highest number of confirmed, deaths, recovered and active cases will be observed in the region It will show significant improvement in recovery cases
	Deaths	10,648	48,307	104,329	
	Recovered	217,681	1,269,646	2,609,589	
	Active	150,327	399,877	996,047	
Maldives	Confirmed	2873	5970	7081	Both the confirmed and recovered cases will increase with a similar trend Death cases will also fall to zero as active cases
	Deaths	14	0	0	
	Recovered	1139	5970	7081	
	Active	1719	0	0	
Nepal	Confirmed	6295	185,528	1,089,821	Confirmed cases will notably increase. However, the death cases will jump to 2955 from zero in the long‐term prediction.
	Deaths	0	0	2955	
	Recovered	1783	99,292	658,568	
	Active	4512	86,236	428,297	
Pakistan	Confirmed	118,775	332,528	497,800	Confirmed, deaths, recovered and active cases will increase gradually Recovered cases will show almost double in the long‐term prediction
	Deaths	2534	7096	10,623	
	Recovered	49,434	254,510	487,176	
	Active	66,806	70,921	588,234	
Sri Lanka	Confirmed	2496	8832	23,151	In the mid‐term and long‐term forecast, active cases will become zero
	Deaths	0	40	106	
	Recovered	1840	8791	23,045	
	Active	655	0	0	

**FIGURE 4:**
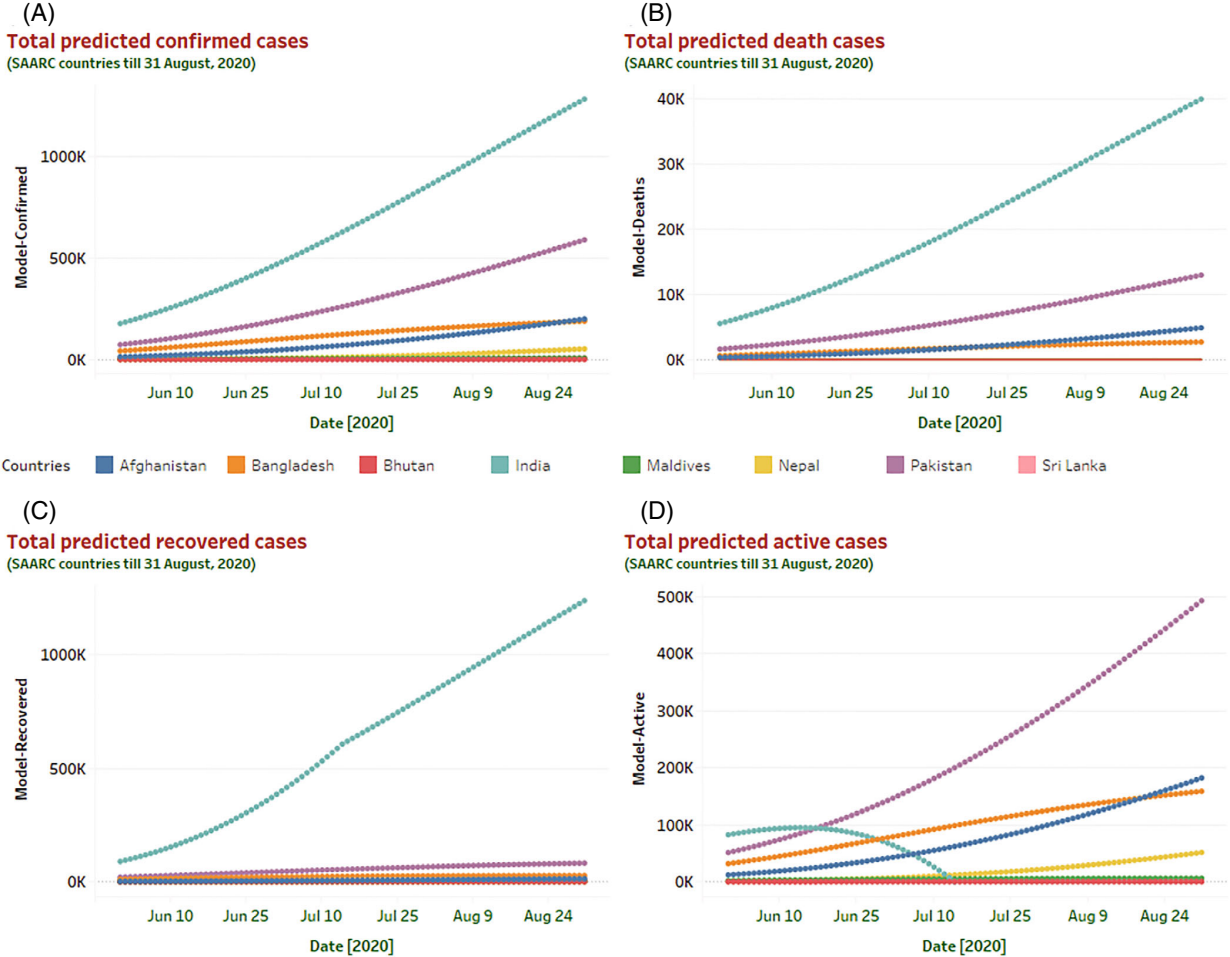
Visualization of a mid‐term prediction curve till August 31, 2020, for all the countries of SAARC regions. Based on the M‐SIR prediction model, prediction statistics for the upcoming 90 days for all the countries in the SAARC regions has depicted here. Different predicted case scenarios (confirmed, death, recovered, and active) till August 31, 2020, help to understand the epidemiological nature of COVID‐19 in the south Asian region. The predicted model indicates that in the next 90 days prediction curve will increase sharply for India (in terms of confirmed and death cases). Moreover, India will also show a substantial increase in its recovery rate. However, the number of active cases will increase in Pakistan till August 31, 2020.

A midterm (90 days) prediction model is further assessed until August 31, 2020, and the nature of the epidemic curve has been depicted in Figure 4. For all the countries of the SAARC regions, we have predicted the total number of confirmed, death, recovery, and active cases.

**FIGURE 5:**
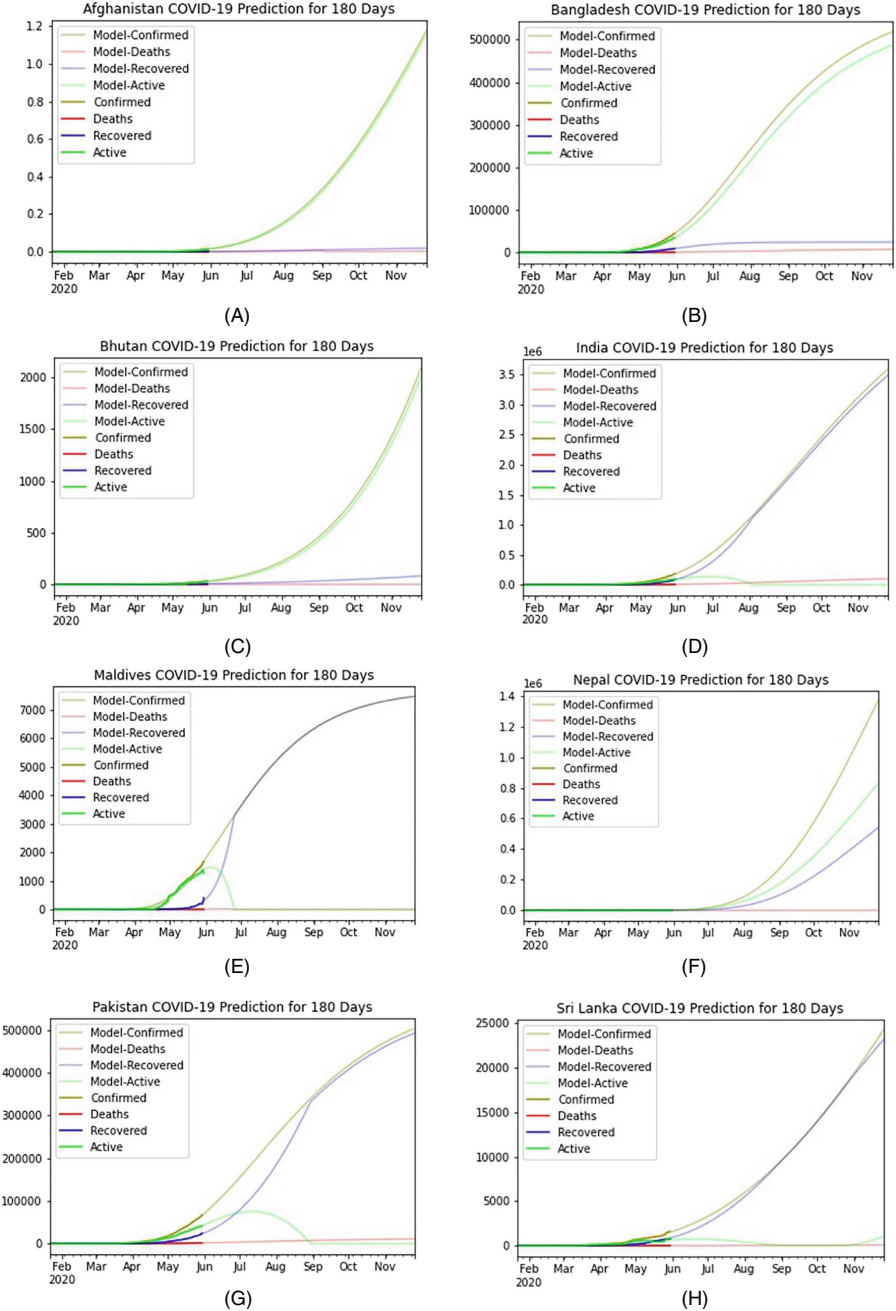
Graphical representation of a long‐term prediction curve till November 30, 2020, for all the countries of SAARC regions. This long‐term prediction model for the next 180 days provides a depiction of all SAARC countries' evaluation of COVID‐19 till mid‐November. All the countries prediction curves are denoted here from (A–H), respectively, where (A) Afghanistan, (B) Bangladesh, (C) Bhutan, (D) India, (E) Maldives, (F) Nepal, (G) Pakistan, (H) Sri Lanka predicted the epidemiological curve for next 180 days successfully.

Lastly, a long term prediction model for the next 180 days (till November 30, 2020) situation has been presented in Figure 5. According to the prediction model, active cases will fall to zero at the end of June in Sri Lanka, and at the beginning of August in India (Figure 5). However, Bangladesh, Maldives, and Pakistan will take more few months to reduce its active cases. On the other hand, countries like Afghanistan, Nepal, and Bhutan will show a steep increase in their active cases.

Since the classic forms of SIR are deterministic, an improved version based on parameter optimization is suggested to improve the prediction. Moreover, all the forecasts showed in the article for the countries of the SAARC region without considering the conditions of quarantine and social distancing. Apart from that, analysis of the data based on available COVD‐19 cases till May 30, 2020. Therefore, the current data of the COVID‐19 case trend is radically different from what this article predicted. With such challenges, the future direction of this research can be extended by employing other epidemiology models (i.e., SEIQR) along with some machine learning (i.e., regression analysis) architecture. Also, consideration of social distancing and quarantine along with other observations including policy actions, human behavior, and restrictions that have the potential to improve forecast accuracy are encouraged for future studies.

## 5 CONCLUSION

The COVID‐19 epidemic has brought unprecedented health concerns for the community all around the globe. This research predicted the epidemic trend of COVID‐19 in SAARC countries on the basis of short‐term, midterm, and long‐term situations. We explored the transmission pattern of a new coronavirus using the M‐SIR compartmental epidemic model, a modified version of the SIR pandemic model. This study also calculated the transmission rate, recovery rate, and reproduction number for eight South Asian countries. In addition, nine different statistical metrics have been analyzed, and it has been determined that Afghanistan, Bangladesh, India, and Pakistan will continue to be the most affected countries in the SAARC area through November 2020. To the best of our knowledge, this study is the very first COVID‐19 prediction model which focused on the countries of SAARC regions. This epidemic modeling can be a helpful tool for estimating and predicting the scale and time course of COVID‐19, evaluation of the effectiveness of public health interventions, and information on public health policies in SAARC countries. In the future machine learning tools can be further used to identify and optimize the time profile for the confinement.

### PEER REVIEW

The peer review history for this article is available at https://publons.com/publon/10.1002/eng2.12550.

### CONFLICT OF INTEREST

The authors declare that there are no conflicts of interest.

## AUTHOR CONTRIBUTIONS

**Samrat Kumar Dey:** Conceptualization (equal); investigation (equal); methodology (equal); software (equal); visualization (equal); writing – original draft (equal); writing – review and editing (equal). **Md. Mahbubur Rahman:** Conceptualization (equal); formal analysis (supporting); methodology (equal); project administration (lead); supervision (lead); writing – original draft (supporting); writing – review and editing (lead). **Kabid Hassan Shibly:** Conceptualization (equal); data curation (equal); investigation (equal); methodology (equal); software (supporting); writing – original draft (supporting); writing – review and editing (supporting). **Umme Raihan Siddiqi:** Investigation (supporting); project administration (supporting); supervision (supporting); writing – original draft (supporting); writing – review and editing (supporting). **Arpita Howlader:** Conceptualization (supporting); formal analysis (supporting); validation (supporting); writing – original draft (supporting).

## Data Availability

The data that support the findings of this study are available from the corresponding author, upon reasonable request.
